# Eye Gaze Technology as a Form of Augmentative and Alternative Communication for Individuals with Rett Syndrome: Experiences of Families in The Netherlands

**DOI:** 10.1007/s10882-015-9455-z

**Published:** 2015-10-19

**Authors:** Gillian S. Townend, Peter B. Marschik, Eric Smeets, Raymond van de Berg, Mariёlle van den Berg, Leopold M.G. Curfs

**Affiliations:** Rett Expertise Centre – Governor Kremers Centre, Maastricht University Medical Centre, PO Box 616, 6200 MD Maastricht, The Netherlands; Institute of Physiology, Research Unit iDN – Interdisciplinary Developmental Neuroscience, Medical University of Graz, Graz, Austria; Center of Neurodevelopmental Disorders (KIND), Department of Women’s and Children’s Health, Karolinska Institutet, Stockholm, Sweden; Division of Balance Disorders, Department of Otorhinolaryngology and Head and Neck Surgery, Faculty of Health Medicine and Life Sciences, School for Mental Health and Neuroscience, Maastricht University Medical Centre, Maastricht, The Netherlands; Faculty of Physics, Tomsk State University, Tomsk, Russian Federation; Nederlandse Rett Syndroom Vereniging, Dutch Rett Syndrome Parent Association, Utrecht, The Netherlands

**Keywords:** Rett syndrome, Communication, Eye gaze technology, Eye tracking, Augmentative and alternative communication (AAC), Support and training

## Abstract

This paper provides a brief report on families’ experiences of eye gaze technology as one form of augmentative and alternative communication (AAC) for individuals with Rett syndrome (RTT), and the advice, training and support they receive in relation to this. An online survey exploring communication and AAC was circulated to 190 Dutch families; of the 67 questionnaires that were returned, 63 had answered questions relating to eye gaze technology. These 63 were analysed according to parameters including: experiences during trial periods and longer-term use; expert knowledge, advice and support; funding; communicative progress; and family satisfaction. 20 respondents were using or had previous experience of using an eye gaze system at the time of the survey, 28 of those with no prior experience wanted to try a system in the future. Following a trial period, 11 systems had been funded through health insurance for long-term use and two families had decided a system was not appropriate for them. Levels of support during trials and following long-term provision varied. Despite frustrations with the technology, satisfaction with the systems was higher than satisfaction with the support. The majority of families reported progress in their child’s skills with longer term use. These findings suggest that although eye gaze technologies offer potential to individuals with RTT and their families, greater input from suppliers and knowledgeable AAC professionals is essential for individuals and families to benefit maximally. Higher levels of training and support should be part of the ‘package’ when an eye gaze system is provided.

## Introduction

The use of eye gaze or eye tracking technologies to support and facilitate communication with individuals with Rett syndrome (RTT) has been increasingly promoted in recent years (Djukic and McDermott 2012; Garrett 2013; Lariviere 2014). It is fair to say it is the current ‘hot topic’ for many families within the global Rett community. Many parents have reported anecdotally over many years that their children with RTT understand more than they are able to demonstrate on assessment or are able to express for themselves, primarily as a consequence of apraxia (Bartolotta et al. 2011; Urbanowicz et al. 2014). This view can sometimes be at odds with professionals who consider that language and cognitive factors also have a role to play. Interest in and a desire to own and use high-tech eye gaze systems is escalating yet the costs are high.

RTT is a rare neurodevelopmental disorder found predominantly in females, characterised by stages of stagnation, regression and (possible) recovery in skills following seemingly near-normal development in the early years of life. A number of authors have written about the staging processes and ages at which they most commonly arise (Charman et al. 2002; Hagberg 2002; Lee et al. 2013; Smeets et al. 2012). Of particular significance to this study is the propensity for individuals with RTT to engage in “intense eye communication” (Neul et al. 2010, p. 946). Other prominent signs contributing to the profile of RTT are stereotypic hand wringing/clapping movements and abnormal gait or an inability to walk. Diagnosis of RTT is primarily clinical, based on the presence or absence of criteria which were revised in 2010 by members of the RettSearch consortium (Neul et al. 2010). Following clinical identification, the diagnosis may then be confirmed by genetic analysis which, in the majority of cases, is attributed to mutations in the Methyl-CpG-binding protein 2 (*MECP2*) (Amir et al. 1999; Christodoulou and Ho, 2001, updated 2012).

Individuals with RTT demonstrate severe limitations in their ability to communicate through conventional channels such as speech and hand signs/gestures (Cass et al. 2003), due in (large) part to the influence of apraxia or an inability to control purposeful, voluntary movement (Djukic and McDermott 2012). In recent years a number of studies have sought to probe deeper into the communicative behaviours of individuals with RTT, to explore levels of underlying intentionality and the range of functions as well as the means by which they are able to express themselves (e.g., Bartolotta et al. 2011; Bartl-Pokorny et al. 2013; Didden et al. 2010; Hetzroni and Rubin 2006; Julien et al. 2015; Marschik et al. 2012; Urbanowicz et al. 2014). In general the findings of these studies suggest that the majority of individuals with RTT communicate at levels labelled as pre-linguistic and/or pre-intentional. However, it may be more appropriate to refer to the restricted range of socio-communicative functions, where behaviours are interpreted by caregivers and communication partners, as ‘potential communicative acts’ (Sigafoos et al. 2000; Marschik et al. 2012). Such behaviours include the use of stereotyped hand movements, facial expressions, body movements, (undifferentiated) vocalisations, eye gaze and even hyperventilation, and are commonly acknowledged to be in order to seek attention, protest, request or make a choice. Several studies also point to apparently low levels of language comprehension and cognitive functioning (Berger-Sweeney 2011), especially when standardised receptive language, IQ or adaptive behaviour tests are employed. Two useful overviews of such studies are given by Demeter (2000) and Sigafoos et al. (2011); both reviews point out the problems associated with reliably assessing individuals with RTT and highlight the need for functional tests which balance observational and standardised measures and which ultimately provide a “gold standard” for assessment (Sigafoos et al. 2011, p.698).

One of the universal features of the above-mentioned studies into communicative ability is the reported use of eye gaze as the most frequent form of expressive communication. Some authors note that eye gaze may simply amount to fixating on an object, raising the question of whether individuals with RTT can use true referential eye gaze, switching between a desired object and a partner (Bartolotta et al. 2011; Cass et al. 2003). Hetzroni and Rubin (2006) were able to show that this behaviour can be trained in individuals with RTT. Other studies exploring cognitive performance through the use of more advanced eye tracking technologies have produced mixed results. For example, the study by Baptista et al. (2006) demonstrated that children with RTT could follow key word instructions, recognise and match picture pairs, and categorise pictures. However, a later study was unable to prove the recognition of concepts of size, colour, shape and position (de Lima Velloso et al. 2009). Undeterred by this ‘failure’, a number of more recent studies (Djukic et al. 2012, 2014; Rose et al. 2013) have fuelled interest in the potential benefits that eye tracking technologies can offer to individuals with RTT and have led to calls for the development of more objective eye tracking-based cognitive and receptive language assessments which can be used to validate data gathered from parental reports (Byiers and Symons 2013; Urbanowicz et al. 2014).

Eye trackers measure the “Pupil Centre Corneal Reflection (PCCR)” (Holmqvist et al. 2011) using a camera to detect the reflection of near infra-red light on the cornea and pupil of each eye in order to determine where the eye gaze is directed. Eye tracking control is potentially life-changing for individuals with severe physical disabilities who are unable to control a computer through more conventional means such as a mouse or keyboard or are unable to utilise other high-tech voice output communication aids (VOCAs) which require access through touch, for example, via a switch or touchscreen. When combined with speech output, eye tracking technology can give users a ‘voice’. For such an application an intact oculomotor system is required. ‘Saccades’, ‘fixation’ and ‘smooth pursuit’ (Fite and Walker 1990; Holmqvist et al. 2011) are crucial for purposeful use of eye gaze/tracking technologies. To date, most of the literature published on this technology as a form of AAC relates to use with client groups other than RTT, such as those with Amyotrophic Lateral Sclerosis. Researchers such as Djukic and McDermott (2012), Djukic et al. (2012, 2014) and Rose et al. (2013) are beginning to publish about the applied use of eye tracking research in relation to RTT and literature promoting the use of, or sharing experiences of using, eye gaze/tracking technologies for intervention, for developing communication skills and/or facilitating day to day communication in RTT is just beginning to emerge (Garrett 2013; Lariviere 2014, 2015; Wandin et al. 2014). However, most reports are anecdotal and shared by families and professionals through social media, workshops and seminars or webinars. Therefore, it seems pertinent to ask questions and to plan for the time ahead. To what extent are such systems being taken up and what are the factors that come into play when doing so? What support needs do families have and are those needs being met? If they are not, how can they be met in the future? These questions are the focus of this study, utilising the Dutch Rett community as a single population case study.

This research focuses on the use of eye gaze/tracking technology from the perspective of families. Research questions are: (1) Are families of individuals with RTT using eye gaze technology to enhance communication? If so, (2) what are the families’ support needs in relation to this and how well are they being met? (3) Do families see progress in their child’s communication skills? (4) What implications for future service delivery and support for communication can be drawn from families’ experiences to date?

## Method

### Participants and Survey Tool

During the summer of 2014, all 190 families on the mailing list of the Nederlandse Rett Syndroom Vereniging (NRSV, Dutch Rett Syndrome Parent Association) were invited to participate in an online survey relating to communication skills and families’ experiences of communication support. The survey was developed by the Rett Expertise Centre Maastricht using Qualtrics online survey software as the host-platform (see http://www.qualtrics.com/). Embedded within the broad survey was a special focus on eye gaze technology.

### Analysis

The survey responses were subjected to multi-variant qualitative analysis using the statistical analysis and data reporting tools contained within the Qualtrics software. The results are presented as descriptive statistics. It should be noted that the number of individuals responding to each question was not consistent; no questions were compulsory and individuals could choose not to answer a question if they wished. Thus, not all respondents answered all questions that were open to them.

## Results

Sixty-seven out of 190 families completed and returned a questionnaire (return rate = 35 %), representing individuals with RTT ranging in age from 3 years 6 months to 60 years 6 months. 63/67 families responded to the questions asking whether their child used now, or had used in the past, an eye gaze system. 20/63 (age range 3 years to 18 years 6 months) reported experience of using a system and 43/63 had never used one.

### Trial Periods, Applications for Funding and Provision of an Eye Gaze System

**Fig. 1:**
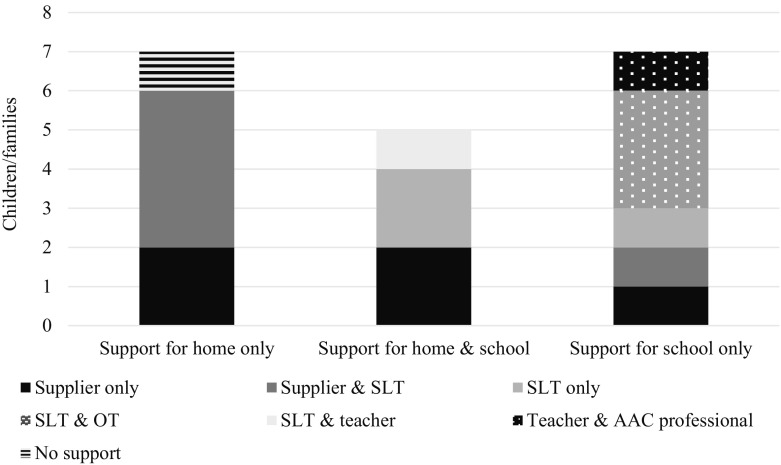
Support during trial periods (*n* = 19)

Of the 20 children with experience, 19/20 were currently involved in a trial or had previously had a trial period with an eye gaze system. Only 1/20 had long-term access to an eye gaze system in school without having had a previous (short-term) trial. Duration of trial periods varied greatly, with the shortest being one week (1/19) and the longest more than three months (2/19). The majority were for 4 (12/19) or 6–8 weeks (3/19). One was of unspecified duration. There was a pretty even split between settings in which trials took place with 7/19 at home only, 7/19 at school only and 5/19 across both settings. Data regarding the levels of support provided during the trials, and by whom, are shown in Fig. 1. The most common form of support reported was that of the supplier offering an introductory session to demonstrate how the system worked and/or to programme the device. Where there was Speech & Language Therapy (SLT) input, alone or with another professional, this usually took the form of one or two sessions in school each week (further details on what constituted a ‘session’ were not given).

5/19 children were engaged in a trial at the time of the survey. Of the 14/19 whose trial had ended, 12/14 families had submitted a request for funding of a system to their health care insurer while 2/14 had decided that an eye gaze system was not appropriate as their child was too mobile or suffered from too many seizures. One family was still awaiting a decision on their application but the remaining 11/12 requests had been honoured in full.

Of the 43 families who had not yet tried an eye gaze device, 28/43 expressed a desire to arrange a trial period in the near future; 23/28 of these individuals were under the age of 25. Of the 13/43 who were not interested in trialling eye gaze technology, 10/13 were over the age of 25. 2/43 families did not say whether they wanted a trial.

### Long-Term Experiences of Using an Eye Gaze System (Post-Provision)

**Fig. 2:**
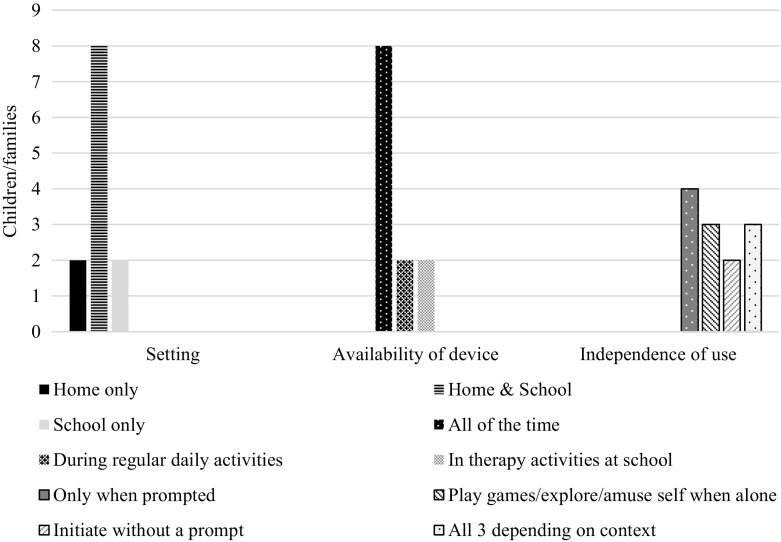
Setting, availability of device and independence with long-term use (*n* = 12)

In total, 12 children had access to an eye gaze system for long-term use, 7/12 had been using theirs for 6–12 months, 3/12 for 3–6 months, 1/12 for less than a month and 1/12 for more than a year. The settings in which each child was using their system, the availability of the device and how independent the child was reported to be in using it are shown in Fig. 2. The range of people with whom each child used their system reflected the settings in which the systems were being used, that is, with family members, therapists, teachers/caregivers and other familiar people. The devices were more likely to be available throughout the day at home than at school, and were mounted on a wheelchair, table, or wall in the case of a child who was ambulatory.

**Fig. 3:**
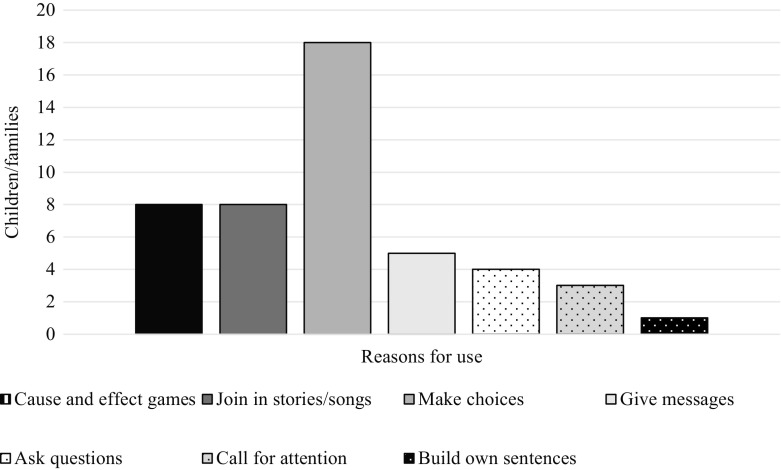
Reasons for use/communicative functions (*n* = 11)

11/12 families shared information on the reasons for which their child used their eye gaze system (for example, the communicative functions they conveyed) and the type of page-sets used. Most children used custom-made page-sets with a combination of photos and graphic symbols (8/11), although 1/11 reported using custom-made pages with photos only and 1/11 with graphic symbols only while 1/11 used a combination of custom-made and pre-designed (‘off-the-shelf’) page-sets with both photos and symbols. All children used their devices for more than one reason, for example, to engage in cause and effect games and to make choices, as illustrated in Fig. 3.

Reports of ongoing support varied. Of the 9 families who answered questions relating to post-provision support, 4/9 received one or two home visits from the supplier to set up the device/train them in its use when it was first delivered, 4/9 received (varying amounts of) support from an SLT and/or class teacher in school or day centre and 1/9 employed an SLT to work with their child at home. When it came to programming devices and adapting vocabulary/adding words so that is was appropriate for the child’s needs, 9/9 parents reported that they themselves were responsible for this, although carers within school gave some support in one case.

### Progress

Eleven families responded to questions relating to progress in communication skills. 10/11 felt that they could see progress in their child’s levels of awareness and engagement and their ability to express themselves since having an eye gaze system. They also reported an (apparently) increased understanding of language. One parent qualified this by saying that progress was slow; only 1/11 claimed to see no progress at all.

### Family Satisfaction

**Fig. 4:**
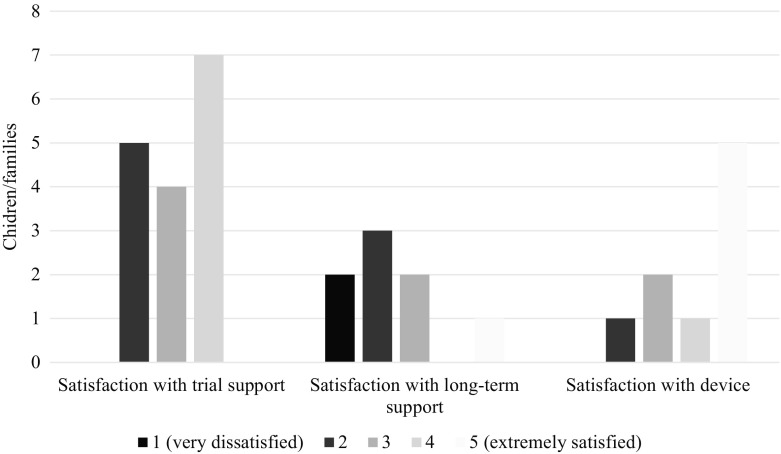
Satisfaction with support during trial period and long-term use, and with the device (n = variable)

Family satisfaction was explored across three domains: with support during the trial period, with support for long-term use (post-provision) and with the technology itself. The levels of satisfaction can be seen in Fig. 4.

When asked what could be improved across the whole trajectory, responses included: more ‘hands-on’ and intensive support from the supplier, both with planning and preparation before and during trial periods and with long-term provision; longer trial periods and trials of a greater range of devices/systems; increased knowledge and understanding of how to use the technology among therapists and other professionals, such that they could provide a higher level of support to the child and to the family; and (low-budget) training workshops for families. Technical improvements such as a faster processor and more reliable hardware were also requested. In addition, respondents recognised the value of self-help group(s) and network(s) and requested that a joint position statement be issued by the national parent association and Rett Expertise Centre setting out optimal levels of support recommended to maximise use of, and benefit from, an eye gaze system.

## Discussion and Conclusion

The results of the Dutch survey offer some insight into how eye gaze technologies are beginning to be used within the Rett community. There were some frustrations with the technology itself but, on the whole, families who had experience of an eye gaze system reported that they were happy with it, could see progress in their child’s skills (depending upon the length of time they had had a device) and were very positive about the potential offered.

Although the number of families who reported on their experiences of the technology at the time of the survey was relatively small, it is worth noting that since the survey many of the families who expressed a wish to trial an eye gaze system have indeed done so and the number of families submitting applications to their health care insurer and subsequently provided with a system for long term use has grown substantially. It is no longer the case, however, that all requests are honoured by insurance companies and subsequent to the survey more questions are being raised by insurers about the potential of individuals with RTT to benefit in the face of the high cost of eye gaze technology. This highlights the need articulated in this and other studies (e.g., Townend 2007) for well-conducted, well-supported trials over a longer period of time, or even the creation of technology loan banks from which expensive equipment can be loaned and recycled/exchanged more easily if it proves to be inappropriate after a period of well-supported use or as needs and circumstances change. In addition, there is a role for the development of thorough assessment techniques which take into account not only motivational and affective factors and cognitive and receptive language abilities, but also explore the little-researched function of the oculomotor system in individuals with RTT and the potential effects of apraxia and delayed reaction time on the ability to use eye tracking technology with purpose. Such assessments are especially relevant in cases where an individual is struggling to utilise an eye gaze system effectively, and could contribute to better understanding the mechanisms underlying functional use of eye gaze for communication and help to pinpoint where breakdown may lie.

It is interesting to note the difference between families of individuals under and over the age of 25 expressing interest/disinterest in trialling an eye gaze system. Families were not asked to elaborate further on this preference so underlying reasons can only be speculated, however, this is an issue which could usefully be explored further. By and large, those over 25 lived away from home so families may not have felt able to make a commitment on behalf of carers who were responsible on a daily basis. Yet, informal research in the US and UK indicates that “eye gaze technology is ‘opening up the world’ of communication and literacy learning for individuals with RTT” of all ages (Lariviere 2014, p.4), with examples of women aged 29 (p.9) and 42 (Garrett 2013) navigating their way to successful communication as soon as they were introduced to a system. Introduction of new technologies in adulthood should not, therefore, automatically be closed.

The levels of training and support offered post-provision, as well as during the trials, were highlighted by families as an important factor modulating successful adoption and use of a device. There is a clear need to build the knowledge and skills of everyone in the individual’s social network if complex communication technology is to be used to maximum effect, i.e. therapists, educators, and other carers as well as close family members. This has been clearly demonstrated by surveys of wider client groups using a diverse range of AAC (Judge and Townend 2013; Sutherland et al. 2005) and the use of eye gaze technology within the RTT population is no exception. This need for skill-building can be fulfilled in a multiplicity of ways. At one level, the sharing of knowledge and advice based on personal experiences can take place informally between parents (and professionals) through social media fora (for example, via Facebook groups) and more formally through the organisation of workshops and seminar during national parent association Family Days. At another level, collaborations between national parent associations from different countries and the development of professional networks within and across countries are important avenues for the dissemination of knowledge, skills and support (Townend et al. 2015). At a more individual level, in-depth personalised training and greater hands-on support from suppliers and therapists can be written into the package of care that each family receives with provision of their device. This will naturally carry with it a greater cost. At the current time, within The Netherlands, AAC and therefore eye gaze technology when used to enable communication, falls under the banner of ‘medical equipment’ and is funded by health insurance companies or the AWBZ (Algemene Wet Bijzondere Ziektekosten), a Dutch health law aimed at financing care costs for individuals with long-term chronic illnesses. In certain instances schools or rehabilitation/day centres may purchase their own devices but these will be institutionally-based rather than assigned to one individual. The source of funding notwithstanding, the principle remains the same: training and support costs should be included within the package cost when a device is supplied to an individual. This may increase the price at face value but will help to ensure better value for money by maximising benefit from the system and reducing abandonment through lack of support (Baxter et al. 2012; Johnson et al. 2006).

A frustration experienced by families was the time commitment needed to build vocabularies and page-sets within systems, especially as parents were expected to do this. The majority of parents wanted the eye gaze system delivered with a personalised pre-installed vocabulary, with more challenging software included as part of a learning curve, allowing opportunities for practice and helping parents to monitor their child’s progress. In other countries (e.g. the UK) greater use is made of a range of commercially-produced page-sets which offer a comprehensive starting point, requiring customisation but involving less work than constructing an entire vocabulary from first principles. In The Netherlands, such pre-designed page-sets are less frequently used, perhaps because fewer are translated into Dutch, and families were certainly keen to see this as a development in the future, especially as the difficulty in building up vocabularies was one of the reasons cited for slow progress. The sharing of pages between families is one simple way in which parents can already offer each other support in this area. This can be at a local/national level amongst members of one parent association, for example, and also at a pan-European or international level through collaborations between parent associations of different countries.

As follow-up to the survey, a number of actions are planned: circulation of a further survey to the speech and language therapists and other AAC professionals named by families, to capture their perceptions, knowledge and experience of eye gaze technology, and the needs they identify; creation of therapist support networks relating to RTT and the use of eye gaze technologies; research into oculomotor movement, apraxia and RTT; and a pilot project to test the validity of a receptive language assessment using eye gaze technology as the form of access.

The findings from the survey represent a small population, with a limited response rate, but nevertheless offer a snapshot of the take-up and use of eye gaze technologies in The Netherlands. Eye gaze (eye tracking) technologies offer potential for individuals with RTT and their families. However, greater input from suppliers and knowledgeable AAC professionals is essential for individuals and families to benefit maximally and the development of oculomotor, cognitive, and receptive language assessments utilising eye gaze technology is crucial to demonstrate, if possible, an individual’s underlying and possibly hidden potential.
